# Evolution of a clade of *Acinetobacter baumannii* global clone 1, lineage 1 via acquisition of carbapenem- and aminoglycoside-resistance genes and dispersion of ISAba1

**DOI:** 10.1099/mgen.0.000242

**Published:** 2019-01-16

**Authors:** Mohammad Hamidian, Jane Hawkey, Ryan Wick, Kathryn E. Holt, Ruth M. Hall

**Affiliations:** ^1^​ School of Life and Environmental Sciences, University of Sydney, Sydney, Australia; ^2^​ The ithree Institute, University of Technology Sydney, Ultimo, NSW, Australia; ^3^​ Department of Biochemistry and Molecular Biology, Bio21 Institute, University of Melbourne, Melbourne, Australia; ^4^​ London School of Hygiene and Tropical Medicine, London, UK

**Keywords:** *Acinetobacter baumannii* global clone 1 (GC1), carbapenem resistance, *oxa23*, Tn*2006*, ISAba1, Tn*6168*, AbGRI3, homologous recombination

## Abstract

Resistance to carbapenem and aminoglycoside antibiotics is a critical problem in *Acinetobacter baumannii*, particularly when genes conferring resistance are acquired by multiply or extensively resistant members of successful globally distributed clonal complexes, such as global clone 1 (GC1) . Here, we investigate the evolution of an expanding clade of lineage 1 of the GC1 complex via repeated acquisition of carbapenem- and aminoglycoside-resistance genes. Lineage 1 arose in the late 1970s and the Tn*6168*/OCL3 clade arose in the late 1990s from an ancestor that had already acquired resistance to third-generation cephalosporins and fluoroquinolones. Between 2000 and 2002, two distinct subclades have emerged, and they are distinguishable via the presence of an integrated phage genome in subclade 1 and AbaR4 (carrying the *oxa23* carbapenem-resistance gene in Tn*2006*) at a specific chromosomal location in subclade 2. Part or all of the original resistance gene cluster in the chromosomally located AbaR3 has been lost from some isolates, but plasmids carrying alternate resistance genes have been gained. In one group in subclade 2, the chromosomally located AbGRI3, carrying the *armA* aminoglycoside-resistance gene, has been acquired from a GC2 isolate and incorporated via homologous recombination. ISAba1 entered the common ancestor of this clade as part of the cephalosporin-resistance transposon Tn*6168* and has dispersed differently in each subclade. Members of subclade 1 share an ISAba1 in one specific position in the chromosome and in subclade 2 two different ISAba1 locations are shared. Further shared ISAba1 locations distinguish further divisions, potentially providing simple markers for epidemiological studies.

**Impact Statement**

*Acinetobacter baumannii* infections are among the most difficult nosocomial infections to treat, because of the high incidence of carbapenem and aminoglycoside resistance caused mainly by the spread of specific extensively antibiotic-resistant lineages of successful clonal complexes. Here, we compared the genomes of closely related strains within a recently emerged clade of a globally distributed clonal complex and showed that resistance gene content is an unreliable marker even for very close relationships. In this clade, resistance genes have been both gained and lost or replaced by alternate genes. In one case, a large resistance island has been acquired from a different clonal group. Shared, specific, stable insertion sequence (IS) locations are indicative of isolates with a shared history, but it is rare to be able to trace the dispersion of an IS from the time of its initial entry into a cell. The clade studied here initially acquired ISAba1 as part of an ISAba1-bounded transposon and the locations of ISAba1 copies dispersed from it were highly specific subclade markers. Hence, determining the location of transposons and ISs from genome data is potentially a discriminatory typing method that can inform the epidemiology of clonal spread.

## Data Summary

The finished genome assembly and annotated sequences for *Acinetobacter baumannii* strain A85 were deposited in GenBank under accession numbers CP021782–CP021786 (URLs - https://www.ncbi.nlm.nih.gov/nuccore/CP021782.1, https://www.ncbi.nlm.nih.gov/nuccore/CP021783.1, https://www.ncbi.nlm.nih.gov/nuccore/CP021784.1, https://www.ncbi.nlm.nih.gov/nuccore/CP021785.1, https://www.ncbi.nlm.nih.gov/nuccore/CP021786.1).

## Introduction

Multiple antibiotic-resistant strains belonging to two *Acinetobacter baumannii* clonal complexes have been circulating on a global scale since the mid-1970s. Early isolates of these two globally distributed clones, global clone 1 (GC1) and GC2 (also referred to as European clones I and II or international clones I and II) carried only genes conferring resistance to the early antibiotics (tetracycline, sulfonamides, some aminoglycosides) and further events were needed to confer resistance to current antibiotics, such as fluoroquinolones, third-generation cephalosporins and carbapenems, which were introduced in the 1980s. The latter process resulted in distinct lineages or sub-lineages within each clonal complex [1–3]. Further variation arose from the repeated replacement of genes that determine the structure of the capsule and the outer core of lipooligosaccharide [1, 4–6]. Hence, there is considerable heterogeneity within each clonal complex and the differences can be useful to distinguish close relatives causing outbreaks or dissemination at the local, national or global level.

A previous analysis of 44 GC1 isolates from Australia, Europe and North America for which genome sequences were available revealed two distinct lineages [1]. Most of these isolates (37 of the 44) belonged to lineage 1 and carried at least a remnant of a large AbaR-type resistance island at a specific location in the chromosome. The AbaR island appeared in the mid to late 1970s [1], and all of the antibiotic-resistance genes contained within it [*tetA*(A), *catA1, bla*_TEM_, *aphA1b, aacC1, aadA1* and *sul1*] were acquired in a single step from the resistance region of an M1 plasmid related to R1215 [7]. These genes included ones that conferred resistance to antibiotics that were used therapeutically in the 1960s–1970s (tetracycline, sulfonamides, gentamicin), as well as ones that were of little value due to natural resistances of the species (*catA1*; chloramphenicol resistance). The earliest known GC1 isolates, such as A1 [8], were susceptible to the antibiotics introduced in the 1980s, fluoroquinolones, third-generation cephalosporins and carbapenems, and resistance to each of these had to be acquired separately.

Resistance to third-generation cephalosporins most often arises via increased expression of the intrinsic *ampC* gene caused by the presence of an upstream insertion sequence (IS) that supplies a strong promoter [9, 10]. This can occur either by *de novo* transposition of the IS to this position or by replacement of the *ampC* gene with one from another isolate that has already acquired the upstream IS [11, 12]. Fluoroquinolone resistance requires specific mutations in *gyrA* and *parC* genes, and again this can arise *de novo* or be acquired from another *A. baumannii* isolate that already has the appropriate mutation [1]. Though modest resistance to some of the carbapenem antibiotics can arise via IS activation of specific alleles of the intrinsic *oxaAb* gene [13, 14], a number of carbapenem-resistance genes have now been identified [13, 15] and theoretically these can be found on plasmids or in the chromosome. Most of the carbapenem-resistant GC1 isolates examined carried the most prevalent carbapenemase-encoding gene, *oxa23,* though *oxa58* and *oxa235* (237 variant) were also detected [1].

**Fig. 1.**
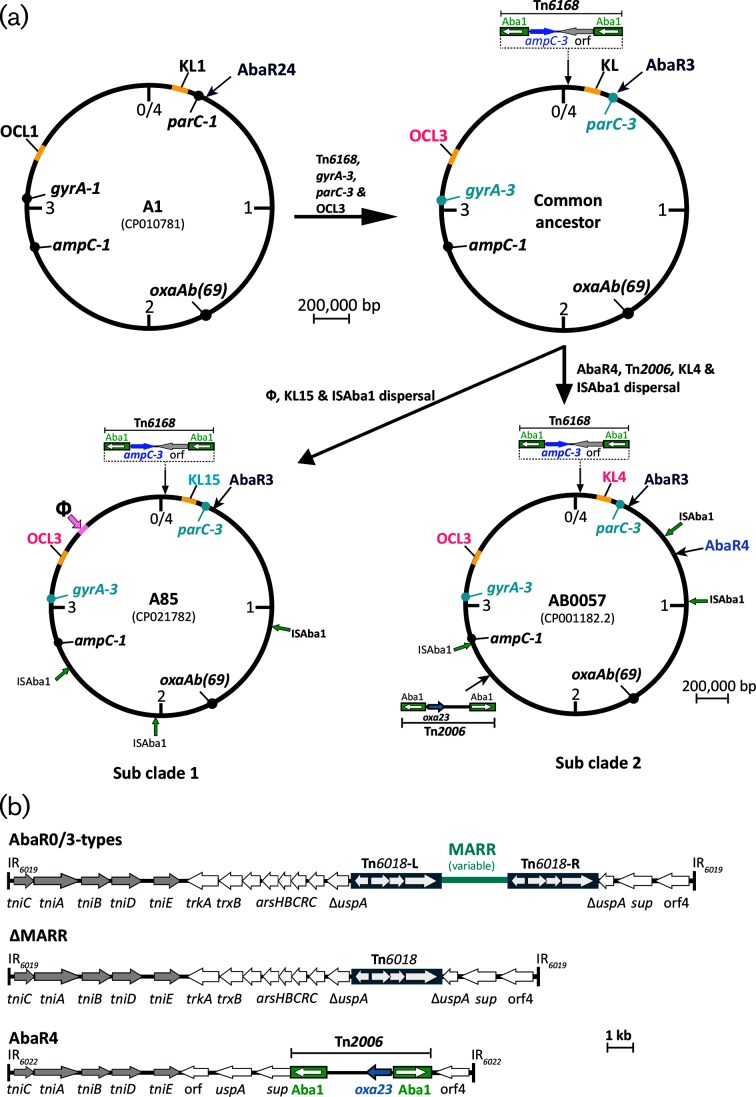
Circular maps of the chromosome of GC1 *A. baumannii* strain A1, an intermediate hypothetical common ancestor, A85 and AB0057 (a) and general structure of the AbaR0/3-type and AbaR4 islands (b). (a) Locations of important features of each chromosome, e.g. *gyrA*, *parC, oxaAb* and *ampC* genes, KL and OCL, and the location of insertions (e.g. Tn*6168*, AbaR, Tn*2006*) are indicated by arrows. Thick green arrows indicate the chromosomal locations of the ISAba1 copies, and a pink arrow indicates the phage region. In subclade 1, only one of the three solo ISAba1 copies found in A85 is shared by all members of the group (shown in bold). In subclade 2, two ISAba1 copies found in AB0057 are present in all members of the group (shown in bold). (b) General structure of the AbaR0/3-type, ∆MARR (Tn*6019*::Tn*6018*) and AbaR4 transposons/resistance islands. The backbones of the AbaR0/3-type islands (Tn*6019*) and AbaR4 (Tn*6022*) are represented by central thick lines bounded by vertical lines indicating the inverted repeats (IR) The extents of genes and ORFs are represented by labelled grey and white arrows. Coloured boxes with internal white horizontal arrows for the genes represent Tn*6018* and ISAba1 copies. The *oxa23* carbapenem-resistance gene is represented by a blue arrow. Features are drawn to scale except for the MARR, which is represented by a cyan line.

Several of the carbapenem-resistant lineage 1 isolates that carried the *oxa23* carbapenem-resistance gene were clustered in a group of 10 isolates that were also resistant to fluoroquinolones and third-generation cephalosporins [1]. Members of this distinct clade shared a set of features that distinguished them from the oldest available GC1 strain, A1 [8], which was used as a reference for the comparative genomic analysis [1]. Isolates in this group had become resistant to third-generation cephalosporins via the acquisition of an ISAba1-bounded transposon, Tn*6168*, carrying a second copy of the intrinsic *ampC* gene [16], which is located at a specific position in the chromosome (Fig. 1). Resistance to fluoroquinolones had occurred via the acquisition of exogenous DNA leading to replacement of the GC1-associated alleles of the *gyrA* and *parC* genes with different alleles, each derived from another *A. baumannii* strain that had already acquired the fluoroquinolone-resistance-determining mutations [1]. Additionally, all members of this clade carried the OCL3 variant at the locus responsible for synthesis of the outer core of lipooligosaccharide instead of the OCL1 variant found in most other GC1 isolates. OCL1 and OCL3 share only six of the nine genes in the OCL gene cluster [4, 6]. The phylogeny indicated that this clade, hereafter referred to as the Tn*6168*/OCL3 clade, is made up of two sub-groups. In one group (hereafter Tn*6168*/OCL3 subclade 1), carbapenem-susceptible isolates were together with a single carbapenem-resistant isolate, A85, which carries the *oxa23* gene in the AbaR4 transposon (transposon Tn*2006* inserted in Tn*6022*; Fig. 1b), which is inserted in an 86 kb RepAci6 plasmid [17]. In contrast, in all members of the second group (hereafter Tn*6168*/OCL3 subclade 2), which includes AB0057, one of the earliest GC1 isolates to have been completely sequenced [18], AbaR4 is in the specific chromosomal location originally seen in AB0057. This indicates that acquisition of resistance to carbapenems is a relatively recent event and is consistent with acquisition of carbapenem resistance after separation of the two groups.

Here, we have examined an expanded set of 27 whole-genome sequences for isolates from Australia, Asia, North America and Germany belonging to the Tn*6168*/OCL3 clade, using as references the completed genome of A85, reported here, and the revised complete genome of AB0057 [19], representing the two subclades (Fig. 1). Various types of diversification, such as the KL type and the context and location of the resistance genes including the *oxa23* gene, were traced. As IS locations have been shown to be informative for tracking the spread of specific *A. baumannii* strains [3, 20–22], the location of all copies of ISAba1 in the *A. baumannii* chromosome of each member of the Tn*6168*/OCL3 clade of GC1 were also identified and mapped.

## Methods

### Bacterial genomes studied

A set of inclusion criteria defined here, i.e. containing Tn*6168*, OCL3, as well as specific alleles of the *gyrA* and *parC* genes (allele 3), was used to screen the GenBank non-redundant database and whole-genome shotgun (WGS) sequence databases to find suitable genomes. In total, 27 GC1 strains (Table 1) were found to share these features, including 3 Australian GC1 strains from our collection. Genome data for six additional previously characterized GC1 strains belonging to different clades were added to the study to serve as references in phylogenetic analysis.

**Table 1.** Properties of GC1 (ST1) *A. baumannii* strains

Strain*	Year	Country	Source	ST_OX_	KL	*oxa23*	AbaR#	GenBank acc. no.	Reference
**A85**	2003	Australia	Sputum	781	15	+	3	CP021782†	[1, 16, 17]
**RBH3**	2002	Australia	Endotracheal aspirate	781	15	−	3	FBXD00000000	[1, 16]
6870155	2002	Australia	Sputum	781	15	−	3‡	JRWO01000000	[40]
**6772166**	2002	Australia	Pus	781	15	−	3	FBWX00000000	[1, 16]
S36	2010	Singapore	nk	491	107§	+	New2||	LAIO01000000	−
NCSR 106	2007	Vietnam	Carriage	491	40	+	31	UCTO01000000	[2]
NCSR 132	2007	Vietnam	Carriage	491	40	+	31	UCTL01000000	[2]
USA15	2013	South Korea	Sputum	491	40	+	10	CP020595†	−
**AB056**	2004	USA	Blood	207	4	+	9	ADGZ00000000	[21]
AB4991	2008	USA	Wound	207	4	+	New1||	LREM00000000	−
MRSN 7339	2004	USA	Wound	207	15	+	3	JPHV01000000	[41]
AR_0045	nk	USA	nk	207	4	+	3	MPBW01000000	−
**AB0057**	2004	USA	Blood	207	4	+	3	CP001182.2†	[18, 19]
1605	nk	USA	nk	947	15	+	3	AUWL00000000	−
TG20277	2006	Germany	Sputum	947	15	+	3	ASFH00000000	−
**Canada-BC1**	2007	Canada	nk	947	15	+	29¶	AMSZ00000000	[41]
**Canada-BC-5**	2007	Canada	nk	947	15	+	29	AFDN00000000	[41]
**AB059**	2004	USA	Blood	207	4	+	3	ADHB00000000	[21]
ABBL051	2008	USA	Blood	207	4	+	∆MARR	LLEF01000000	[42]
**908_13**	2007	USA	Urine	207	4	*+*	∆MARR	AMHW01000000	[43]
**909_02–7**	2007	USA	Sputum	207	4	*+*	∆MARR	AMHZ01000000	[43]
TG22190	2011	USA	Tracheal aspirate	207	4	+	∆MARR	ASFP01000000	−
TG22194	2009	USA	Tracheal aspirate	207	4	+	∆MARR	ASFR01000000	−
TG22196	2009	USA	Blood	207	4	+	∆MARR	ASFS01000000	−
TG22148	2011	USA	Tracheal aspirate	207	4	+	∆MARR	ASFN01000000	−
TG22112	2011	USA	Tracheal aspirate	207	4	+	∆MARR	ASFK01000000	−
TG22214	2010	USA	Sputum	207	4	+	∆MARR	ASFX01000000	−

*Strains in bold discussed in [1].

†GenBank accession number refers to the chromosomal sequence. A85 contains five plasmids, pA85-1 (GenBank accession no. CP021783), pA85-1a (CP021784), pA85-1b (CP021785), pA85-2 (CP021786) and pA85-3 (CP021787). USA15 has one plasmid, pUSA15_1 (CP020594), and AB0057 contains one plasmid, pAB0057 (CP001183.2).

‡Likely to contain AbaR3; contigs making up the AbaR3 island contain string of Ns.

§Similar to KL1 with addition of a *gne1.*

||Numbers not assigned as contigs not assembled.

¶Likely to contain AbaR29; see [1].

#∆MARR is Tn*6019*::Tn*6018*.nk, Not known.

### Complete genome of A85

To close the genome of strain A85 by resolving the complex repeat regions often found in *A. baumannii* genomes, genomic DNA was subjected to sequencing using long-read Pacific Biosciences (PacBio) technology. Genomic DNA was prepared as described previously [1] and subjected to sequencing on two PacBio single-molecule real-time (SMRT) cells (chemistry version C2-P4) at DNA Link (South Korea). A total of 175 588 reads was obtained (GenBank accession no. SRR8181874), with an N50 of 14 964 bp. Reads were combined with available Illumina HiSeq data [1] (ERR110086) using Unicycler [23], which is available at https://github.com/rrwick/Unicycler.

Protein coding, rRNA and tRNA genes were annotated using Prokka [24], and the antibiotic-resistance and polysaccharide-biosynthesis loci, transposons and plasmids were annotated manually. ISs were identified using the ISFinder database, available at https://www-is.biotoul.fr/, and annotated manually.

### Single nucleotide polymorphism (SNP) detection and phylogenetic analyses

Illumina sequence reads were mapped to the A1 reference genome (GenBank accession no. CP010781) using the RedDog pipeline, available at https://github.com/katholt/RedDog. Briefly, RedDog mapped all reads to the reference genome using Bowtie2 v2. 2.9 [25] using the sensitive-local algorithm and a maximum insert length of 2000 bp. Variant sites were called using SAMtools v1.3.1 [26]. Across all isolates, a total of 655 SNPs was identified. SNPs in recombinant regions were removed using Gubbins v2.1.0 with default parameters.

beast v2.4.7 [27] was used to reconstruct a dated phylogeny using the resulting alignment. A relaxed lognormal clock with a uniform prior, and a constant coalescent population model were used. Two samples (1605 and AR_0045) did not have date information, so uniform priors were placed on the dates of these two samples, with the upper bound set to the year they were uploaded to GenBank, and the lower bound set to 1982. Three independent runs were performed, with 50 million iterations each. All runs were combined and subsampled to produce the final maximum clade credibility tree and parameter estimates. Each parameter had an effective sample size (ESS) value >3000.

To ensure that there was temporal structure in the data, the dates for each tip were randomized across the set of sequences, and beast was used to reconstruct a phylogeny using the same model and priors as above. This analysis was replicated ten times, and the resulting mean substitution rates and their 95 % highest posterior density (HPD) for the date randomized alignments were significantly different to the rate estimates using the real data, indicating the presence of a strong molecular clock signal [28].

### Identifying chromosomal locations of ISAba1

Chromosomal locations of ISAba1 were determined using a combination of ISMapper (available at https://github.com/jhawkey/IS_mapper) [ 22], which takes short-read data as input, and ISseeker (available at https://github.com/JCVI-VIRIFX/ISseeker) [ 20], which uses contigs as input, for genomes with no short-read data available at the National Center for Biotechnology Information (NCBI); and where neither program could determine an IS location, manually using the 9 bp direct repeats (DRs) generated by ISAba1 as a guide to pair contigs. Manual assembly was also carried out where the flanking sequences of a given IS were not present in the reference used (strain A85).

To determine the number of ISAba1 gain events across the phylogeny, the presence or absence of each ISAba1 location at each internal node in the phylogeny was determined using ancestral state reconstruction on each ISAba1 site, using the *ancestral.pars* function in the R package phangorn v2.2.0 [29]. Gain events occurred at nodes where the ISAba1 site was present, but the same site was absent at the parent node. A total of 61 gain events was observed across the phylogeny. A linear regression model was used to compare the number of gain events to the amount of time (in years) since the root, for each internal node, and calculate the rate of ISAba1 acquisition.

### Analysis of antimicrobial-resistance loci

All antimicrobial-resistance genes in the genomes were identified by a combination of automated screens, using ResFinder (available at https://cge.cbs.dtu.dk/services/ResFinder/) and manual curation. For the isolates recently found in the GenBank non-redundant and WGS databases, resistance regions were assembled using known resistance regions as templates. AbaR or ∆MARR (∆ multiple antibiotic resistance region) (Tn*6019*::Tn*6018*) regions in *comM* were characterized by retrieving the associated contigs and mapping them to structures established previously as a guide [21, 30–32].

### Analysis of capsule (K) and outer core (OC) loci and regions containing phage sequences

The surface polysaccharide loci were identified by blastn searches for the flanking genes (K, *fkpA*, *lldP*; OC, *ilvE*, *aspS*), as described previously [4, 6]. Each locus was matched against a set of known K loci [4] or OC loci [6]. A new K locus configuration was assigned a number (KL107) using the nomenclature system described previously [4]. The phaster phage search tool [33] was also used to explore genomes to find regions with significant identities to phage genomes.

## Results

### Features distinguishing the *A. baumannii* Tn*6168*/OCL3 subclades

To further examine the differences between the two subclades of the GC1 Tn*6168*/OCL3 clade, the complete sequence of *A. baumannii* strain A85, representing subclade 1, was determined and compared to the recently revised genome of AB0057 [19], representing the second subclade. The chromosome of A85 (GenBank accession no. CP021782) was 4 039 997 bp in length compared to 4 055 148 bp for AB0057 (GenBank accession no. CP001182.2) and 3 909 008 bp for the earliest sequenced GC1 isolate A1 (GenBank accession no. CP010781.1). A85 also contains five plasmids, ranging in size from 2.3 to 86.3 kb (see Fig. S1, available with the online version of this article), the largest of which, pA85-3 (86.3 kb), carries AbaR4 (and hence the *oxa23* gene) and has been described previously [17].

Comparison of the AB0057 and A85 chromosomes using brig [34] confirmed that AbaR4 and an additional copy of Tn*2006* found in the AB0057 chromosome (Fig. 1a) were not in the A85 chromosome, and revealed a large insertion in A85 that was not present in AB0057 (Fig. 1a). This insertion of 32 310 kb (bases 3 479 311–3 511 622 in CP021782.1) was identified as a potential integrated phage genome using phaster [33] with an *int* gene encoding a site-specific recombinase at one end. As noted previously [1], the gene clusters present at the K locus were different (KL4 in AB0057 and KL15 in A85). Two copies of ISAba13 and one of ISAba26 found in the AB0057 chromosome [19] are in the same positions in the A85 chromosome and, hence, are likely to have been present in the common ancestor.

### ISAba1 entered the clade with Tn*6168*

Many GC1 isolates do not include ISAba1 [1, 16], but it has entered various lineages of this clone. Adams and co-workers previously examined the locations of all copies of ISAba1 in the AB0057 chromosome [21] and the locations of the four single ISAba1 copies found are shown in Fig. 1a. However, ISAba1 was not found in any of these positions in A85. Instead three different locations were found (Fig. 1a). The fact that the only shared copies of ISAba1 are those that bound Tn*6168* indicates that ISAba1 entered the chromosome of the common ancestor (Fig. 1a) with Tn*6168*, and then dispersed.

### Further Tn*6168*/OCL3 clade members

**Fig. 2.**
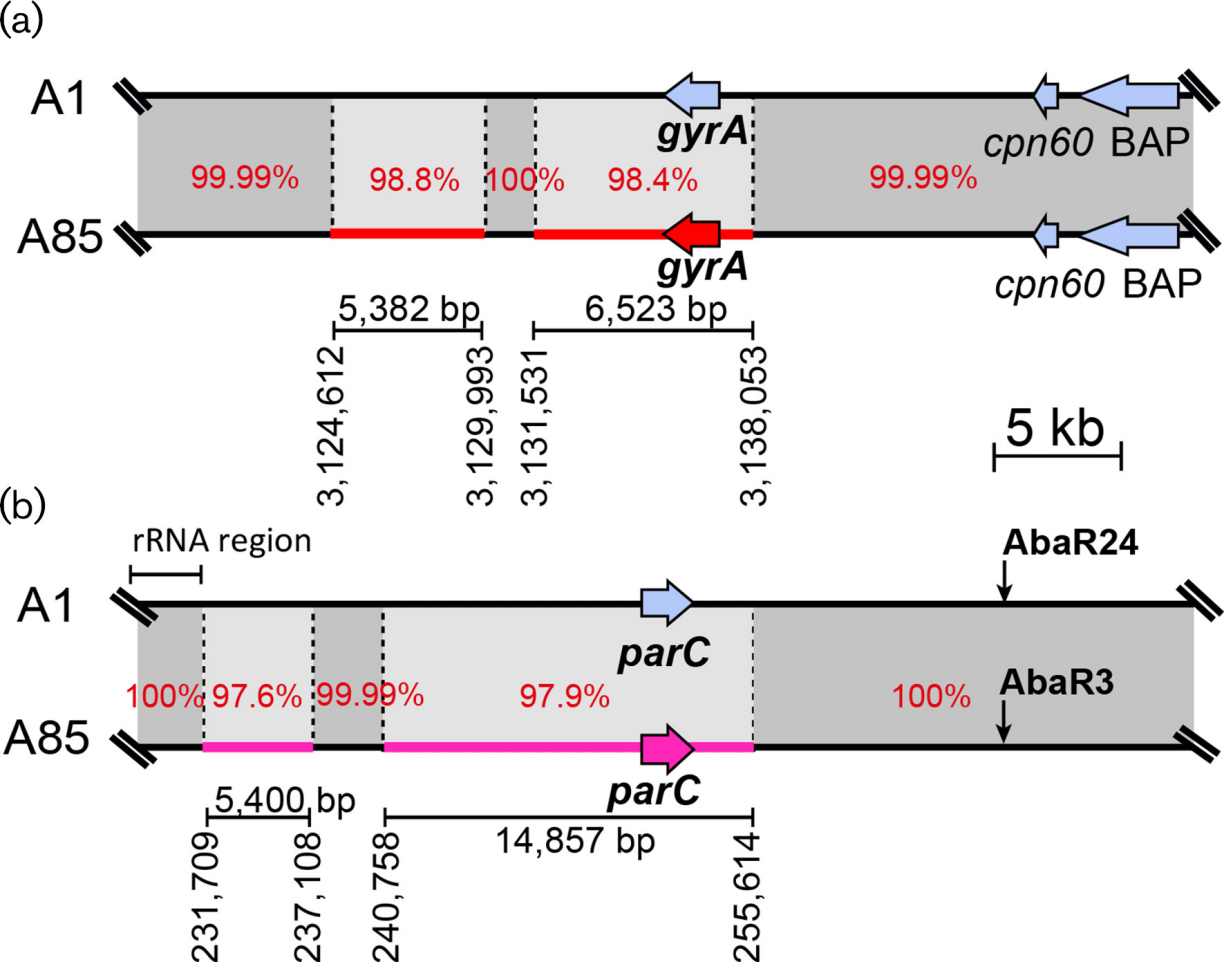
Comparison of the region surrounding (a) the *gyrA* gene and (b) the *parC* gene. The thick black lines represent the 40 kb chromosomal region surrounding these genes in strains A1 (GenBank accession no. CP010781) and A85 (GenBank accession no. CP021782) with red and pink patches indicating imported regions in A85. The locations of AbaR are indicated by vertical arrows. The percentage identities in the patch regions are indicated. The red and pink horizontal arrows indicate the orientation and extent of the *gyrA* and *parC* genes, respectively. The lines below show the extent and positions in the A85 chromosome (GenBank accession no. CP021782) of the patches with the length in bp indicated.

The GenBank non-redundant and WGS databases were searched for further GC1 genomes that are likely to fall into the Tn6*168*/OCL3 clade by seeking evidence first that Tn*6168*, a rare transposon, is present and is in precisely the same chromosomal location as found in AB0057 and A85, and then that the outer core locus was OCL3. Seventeen additional genomes, expanding the set to include isolates from Asia, were found in WGS databases and the properties of the 27 strains studied are presented in Table 1. Comparison of 50 kb on either side of the *gyrA* gene and the *parC* gene in AB0057 and A85 to the corresponding regions in A1 revealed that the recombination patches carrying the introduced *gyrA* and *parC* alleles were 6.5 kb for *gyrA* and 14.8 kb for *parC* (Fig. 2). The same segments were found in the remaining genomes.

Searches of the DNA sequence databases (nucleotide and WGS) revealed that the AB0057/A85 *gyrA* and *parC* alleles were unique. Consequently, the source of the replacement regions could not be identified.

**Fig. 3.**
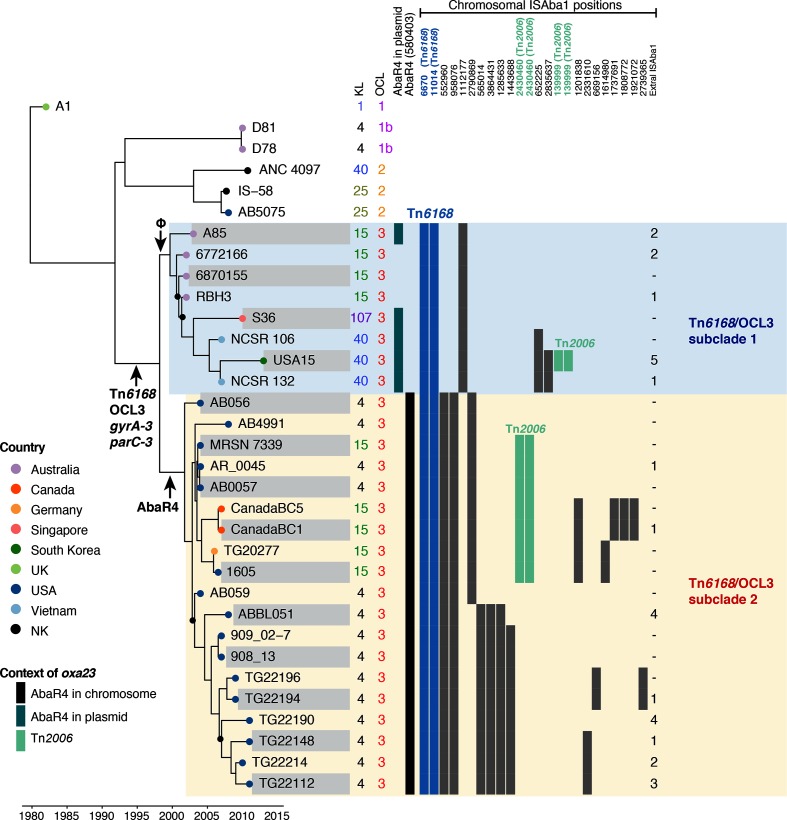
Temporal and phylogenetic analysis of 27 *A. baumannii* GC1 isolates showing the chromosomal distribution of ISAba1 and the surface polysaccharide loci, KL and OCL. Dated whole-genome phylogeny reconstructed using beast with the *x*-axis indicating calendar years. Nodes with black dots indicate a posterior support of <0.7 and all other nodes have support of >0.9. The tree tips are labelled with isolate names and the tip colours indicate the countries, with the key in the left corner of the figure. The KL and OC types are coloured to highlight those that differ from the inferred ancestral types KL1 and OCL1. The first two columns, on the right side of the tree, indicate KL and OCL types, followed by two columns showing the context of the *oxa23* gene, with the key in the bottom left corner of the figure. The remaining columns indicate the presence/absence of ISAba1 copies in given chromosomal positions, with numbers representing the first base of the 9 bp target site duplication generated by ISAba1 insertion (numbers are normalized according to the A85 chromosome, GenBank accession no. CP021782). ISAba1 copies belonging to Tn*6168* and Tn*2006* are shown blue and green, respectively. Small black arrows pointing to different parts of the tree indicate the acquisition of AbaR4, Tn*6168*, OCL3, *gyrA-3*, *parC-3* and phage.

The additional Tn*6168*/OCL3 clade isolates were allocated to a subclade via the presence of AbaR4 at the location seen in AB0057 (12 isolates) or the phage genome at the location seen in A85 (5 isolates), and this allocation was consistent with the phylogeny generated by detailed examination of the 17 new genomes together with the 10 identified previously, using comparative genomic approaches to study the microevolution of their chromosomes. The phylogeny indicated that all of the 27 strains selected on the basis of carrying OCL3, Tn*6168* and the novel *gyrA* and *parC* alleles were clustered into the two subclades (Fig. 3), with isolates from Australia and Asia in subclade 1 and isolates from North America (plus 1 from Germany) in subclade 2.

In contrast to the conservation of the shared features, the gene cluster at the K locus varied within each subclade (Table 1, Fig. 3), as observed previously [1], indicating that replacement of this region of the chromosome has occurred relatively often in recent times (at least twice in each subclade since their most recent common ancestors circa 2000–2002). As a consequence of this and other variations, the sequence type in the Oxford multilocus sequence typing scheme, which uses the *gpi* gene found in the K locus, also varied (Table 1).

### Antibiotic-resistance genes – loss and gain

**Fig. 4.**
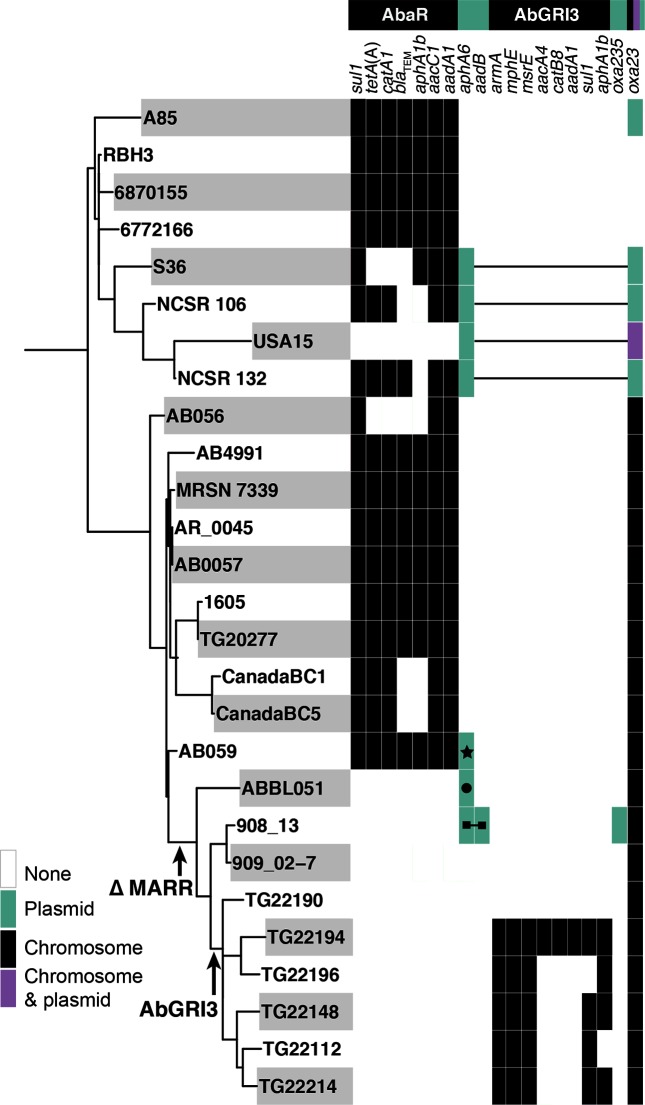
Phylogenetic tree combined with the distribution of antibiotic-resistance genes. Black rectangles indicate resistance genes found in the chromosome, green rectangles represent plasmid-associated genes with symbols indicating different plasmids, and purple rectangles show genes found both in the chromosome and the plasmid in USA15. Antibiotic-resistance genes predicted to be in AbaR and AbGRI3 are separated and shown as two different blocks. The black lines, linking green/purple rectangles, indicate genes found on the same plasmid.

The phylogeny of the Tn*6168*/OCL3 clade was correlated with antibiotic-resistance-gene carriage (Fig. 4). The common ancestor of the Tn*6168* clade appears to have carried AbaR3, which includes *tetA*(A), *catA1, bla*_TEM_, *aphA1b, aacC1, aadA1* and *sul1* genes, located in the *comM* gene (Fig. 1), as this form is seen in several isolates including AB0057 and A85 [17, 19, 35]. However, though all of the isolates examined here carry an AbaR island, some have lost part of the central MARR leading to loss of resistance genes (Fig. 4). Isolates that have completely lost the MARR (indicated by ∆MARR in Fig. 4, Table 1) retain only the Tn6*019*::Tn*6018* backbone (∆MARR in Fig. 1b).

Plasmids have contributed to the resistance-gene arsenal. In addition to A85, four subclade 1 isolates carried the *oxa23* carbapenem-resistance gene. Though in all cases, *oxa23* was in a copy of AbaR4 (i.e. within Tn*2006*, which in turn is within Tn*6022*) that is located in a RepAci6 plasmid, the plasmid was not the same as pA85-3 [17]. An approximately 96 kb plasmid found in USA15, NCSR-106, NCSR-132 and S36 (all of Asian origin), and represented by pUSA15_1 (GenBank accession no. CP020594), has a backbone that is >99.9 % identical to the backbone of pAb-G7-2 (GenBank accession no. KF669606 [35]), but includes an extra 9330 bp next to copy 1 of the short repeat found in RepAci6 plasmids [35]. pUSA15_1 carries the Tn*aphA6* amikacin- and kanamycin-resistance transposon in the same location as in pAb-G7-2, but also carries AbaR4 surrounded by a duplication of bases 43,974–8 of pAb-G7-2. The four isolates carrying this plasmid are clustered in the phylogeny and, hence, the plasmid was acquired by a shared ancestor. In USA15, the Tn*2006* has also migrated from AbaR4 to the chromosome and the MARR has been lost.

**Fig. 5.**
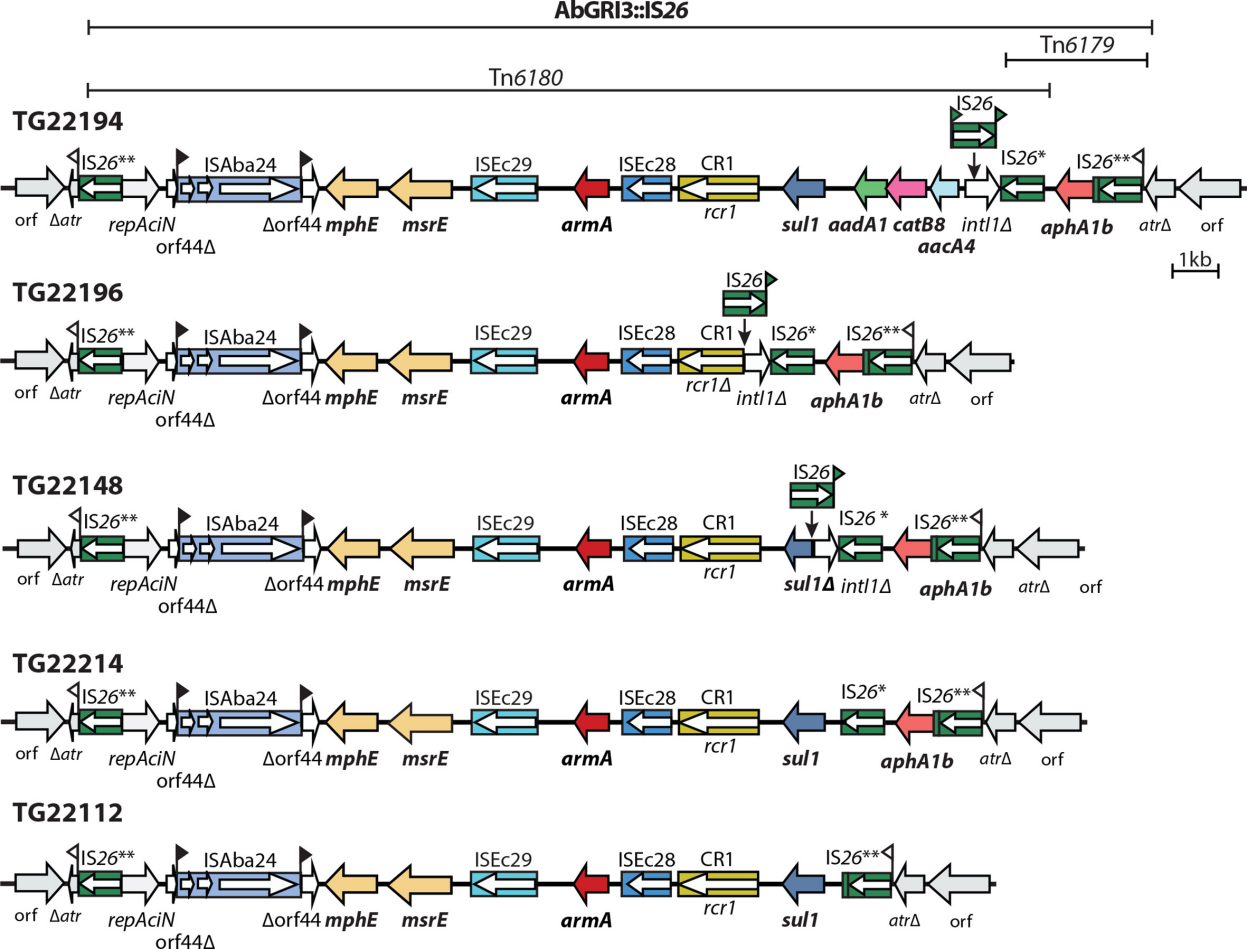
Genetic structure of AbGRI3 variants found in five GC1 isolates. Arrows show the extent and orientation of the genes and antibiotic-resistance genes are in bold. IS and CR (common region) elements are depicted as boxes with their names/numbers indicated above. Arrows inside the IS boxes show the orientation of the *tnp* transposase-encoding gene. A single asterisk indicates the IS*26* is different to the standard sequence of IS*26* at three positions, and two asterisks indicate two differences. Flags indicate 8 bp target site duplications.

In the ∆MARR group of subclade 2, resistance to aminoglycoside antibiotics has been regained. ABBL051 carries Tn*aphA6* in a RepAci6 plasmid that is identical to pAb-G7-2 (35), and 908_13 carries pRAY*, which includes the *aadB* gene cassette (gentamicin and tobramycin resistance) [36] with Tn*aphA6* inserted within it. Surprisingly, in one set of six isolates, the TG22nnn group (Fig. 4), which come from the same study, the strains have acquired the AbGRI3 island, which is bounded by copies of IS*26* (Fig. 5) and carries the *armA* gene (confers resistance to all therapeutic aminoglycosides [37]) in Tn*6180* and the *aphA1* (kanamycin and neomycin resistance) gene in Tn*6179* [38]. AbGRI3 is usually seen in a specific chromosomal location in a post-2000 lineage of the GC2 clonal complex [3, 38]. Inspection of the sequence of the chromosome surrounding the AbGRI3 in the GC1 isolates revealed that it is identical to the corresponding region in the GC2 genome with 34 kb on the left and about 500 bp on the right clearly derived from a GC2 isolate. Hence, this 21 788 bp resistance island has been acquired by importing part of the chromosome from an AbGRI3-carrying GC2 isolate and incorporating a >56 kb segment into the GC1 chromosome via homologous recombination occurring in the chromosomal segments flanking AbGRI3. Variation within AbGRI3 has arisen subsequently, mainly via the action of IS*26* (Fig. 5). Indeed, in TG22190, one member of this group, the AbGRI3 and associated resistance genes have been lost together with some flanking chromosomal DNA, leaving only a single copy of IS*26* and a GC2-derived segment to mark its former presence. As a consequence, this isolate again retains no resistance genes other than *oxa23* in the chromosomal copy of AbaR4.

### Tracking ISAba1 movement

ISAba1 first entered the common ancestor of the Tn*6168*/OCL3 clade with Tn*6168* (see above). Because ISAba1 is not lost from its original location when it moves to a new location, both the IS in the original location and the results of subsequent IS insertion events will be vertically transmitted. Hence, the distribution of ISAba1 insertions in the chromosome was examined (see Methods) in order to find specific markers to differentiate the GC1 Tn*6168*/OCL3 subclades studied here from one another, as well as from the other GC1 clades. The distribution is correlated with the phylogeny for the Tn*6168*/OCL3 clade in Fig. 3.

The locations (given by A85 chromosomal coordinates) of all copies of ISAba1 are in Table S1, and copies that are shared by two or more isolates, in addition to those found in Tn*6168* and bounding Tn*2006* in AbaR4, are shown in Fig. 3. In subclade 1, only one of the three solo ISAba1 copies found in A85 (at 1 112 177 in A85, bold in Fig. 1) is shared by all members of the group and an ISAba1 is not found in this position in subclade 2. In subclade 2, two ISAba1 found at positions 553 764 and 976 679 in AB0057 (552 960 and 958 076 in A85, bold in Fig. 1) are present in all members of the group. Further subdivisions can be seen, such as the ISAba1 at 652 225 in A85 shared by the three members of subclade 1 that also carry KL40. In subclade 2, two further subgroups are distinguished by the presence of either a single ISAba1 at position 2 790 669 (A85 numbering) or three ISAba1 insertions at 565 014, 386 4431 and 1 285 633. Hence, tracking the location of selected copies of ISAba1 is highly discriminatory and should prove valuable in identification of specific GC1 lineages as they spread locally or internationally.

### Plasmids found in A85 and related strains

To examine the persistence of individual plasmids within this group of closely related isolates, other members of the Tn*6168*/OCL3 clade were examined for the presence of the small plasmids found in A85. pA85-2 (8731 bp; GenBank accession no. CP021786) is identical to the RepAci1 plasmids pAB0057 (CP001183.2) and pA1-1 (CP010782), the only plasmid found in AB0057 and A1, respectively. It was also found in all but four isolates (Table 2). It is also very common in the remainder of the GC1 collection examined previously (unpublished observations) but whether this plasmid confers some advantage, perhaps due to the presence of the *tonB* gene encoding a TonB receptor (see Fig. S1), is not known.

**Table 2.** Distribution of small plasmids found in A85 in strains studied here

Strain	pA85-1a (2343 bp) CP021784	pA85-1b (4484 bp) CP021785	pA85-1 (2726 bp) CP021783	pA85-2 (8731 bp) CP021786
A85	+	+	+	+
6772166	+	−	−	+
6870155	−	−	−	+*
RBH3	+	−	−	+
S36	+	−	−	+
NCSR 106	+	−	−	+
USA15	−	−	−	−
NCSR 132	+	−	−	+
AB056	+	−	−	+
AB4991	−	−	−	+
MRSN 7339	+	−	−	+
AR_0045	−	−	−	+†
AB0057	−	−	−	+
Canada-BC1	−	−	−	−
Canada-BC-5	−	−	−	+
TG20277	−	−	−	+
1605	−	−	−	+
AB059	−	−	−	+
ABBL051	−	−	−	+‡
909_02–7	−	−	−	+‡
908_13	−	−	+§	+‡
TG22196	−	−	+	+
TG22194	−	−	+	−
TG22190	−	−	+	+§
TG22148	−	−	+	+§
TG22214	−	−	−	+
TG22112	−	−	−	−

*The plasmid sequence is in nine contigs and bases corresponding to bases 2971–3004 of pA85-2 are missing.

†Bases corresponding to bases 1057–1606 of pA85-2 are missing.

‡The plasmid sequence is in multiple contigs.

§Part of a 15 kb contig.

Other small A85 plasmids found are also listed in Table 2. pA85-1b was not found, but two other small plasmids were seen in a number of further isolates. The smallest, pA85-1a, we found in several of the subclade 1 isolates, and pA85-1 was in a few subclade 2 isolates. Though the sequences of these very small plasmids could have been binned as part of the assembly process for some draft genomes, vertical transmission of these plasmids appears to be less reliable than transmission of ISAba1 copies and the plasmid complement is not a reliable measure of relatedness.

### Evolutionary rate and divergence dates

A dated phylogeny (Fig. 3) was inferred from the alignment of 655 SNPs not associated with recombination events (see Methods) using beast [27]. A1, the oldest GC1 genome available, and five additional genomes of GC1 lineage 1 strains from outside the Tn*6168*/OCL3 clade were included in the analysis to provide context. We estimated the substitution rate to be 1.26×10^−6^ nucleotides per site year^-1^ (95 % HPD 1.0×10^−6^ – 1.5×10^−6^), similar to previous rate estimates in *A. baumannii* [1]. Date randomization tests, as described in [28], indicated that there was strong temporal structure in the data (Fig. S2). The divergence date for GC1 lineage 1 estimated from this data is 1982 (95 % HPD 1977 – 1984), similar to that estimated previously [1]. We estimate the common ancestor of the Tn*6168*/OCL3 clade, which carried Tn*6168*, OCL3 and novel *gyrA* and *parC* alleles, existed in approximately 1998 (95 % HPD 1996 – 2001) (Fig. 3), and had diverged into the two distinct subclades by 2000–2002.

In order to estimate the rate at which stable IS copies accumulate in the chromosome, we used linear regression to model the relationship between branch lengths in the dated phylogeny (which represent years) and the number of inferred ISAba1 acquisition events on each branch (see Methods). This yielded a coefficient of 0.531 (R^2^=0.41, *P*=3×10^−7^); hence, we estimate that, since the initial acquisition of ISAba1 in around 1998 in the common ancestor of Tn*6168*/OCL3, members of this group have accumulated additional stable copies of ISAba1 at a rate of around one every 2 years (Fig. S3).

## Discussion

The published analyses of *A. baumannii* global clones, GC1 and GC2, generally focus on carbapenem-resistance genes and rarely detail the many other significant events that are involved in their ongoing evolution. Our analysis of the Tn*6168*/OCL3 clade of lineage 1 of the GC1 clonal complex has addressed this missing detail and highlights how variable the resistance gene content is, even among the most closely related isolates. However, isolates in this clade all share the Tn*6168* that contributes resistance to third-generation cephalosporins, and the *gyrA* and *parC* genes with fluoroquinolone-resistance-determining mutations that have each been acquired from another *A. baumannii* strain. In contrast, though all of the carbapenem-resistant isolates have *oxa23* in AbaR4, the AbaR4 is located in the chromosome in sublineage 2 but in plasmids in sublineage 1. We have previously emphasized the importance of tracking not only the *oxa23* gene but also which transposon it is part of and where that transposon is located [39], and the analysis reported here supports this conclusion.

Some isolates in both sublineages include the AbaR3 resistance island located in the *comM* gene in the chromosome and have a full complement of genes conferring resistance to early antibiotics, including tetracycline and gentamicin. However, other isolates have a derivative of AbaR3 that has lost some (various AbaR) or all (∆MARR) of these genes. The loss of resistance genes can lead to renewed utility for antibiotics such as tetracycline that are not currently used routinely for the treatment of *A. baumannii* infections. However, in several cases where gentamicin resistance was lost, a different gene conferring resistance to aminoglycosides (*aphA6*, *aadB* or *armA*) has been gained either on a plasmid or, more surprisingly, as part of the AbGRI3 island previously found in GC2 isolates and imported from a GC2 isolate.

Our analysis of the dispersal of ISAba1 in this GC1 clade, which only relatively recently acquired a copy of ISAba1, suggests that fixation of newly transposed copies occurs at a rate of ~1 per 2 years. ISAba1 locations are extremely stable and the locations shared by two or more isolates indicate shared ancestry. They could be lost occasionally if the surrounding segment of the chromosome is replaced by DNA from an exogenous source, but such homologous recombination events can be detected via an elevated SNP density. Recombination between two directly oriented IS copies can also occur if the intervening chromosomal segment that is lost includes no essential gene. As both routes to loss would be expected to be rare, the ISAba1 locations are discriminatory enough to serve as phylogenetically informative markers for tracking the global dissemination of specific lineages and sublineages of the dominant GC1 clones that arise as their evolution continues. Similarly, this approach can be potentially be used to define lineages of the GC2 clonal complex with shared histories. Consequently, either PCR assays or searches of genomic data targeting the most discriminatory ISAba1 locations could simplify the analysis of the epidemiological studies of outbreaks involving multiple closely related types.

The geographical distribution of the two Tn*6168*/OCL3 sublineages examined here warrants further investigation, as currently sublineage 1 is confined to Australia and Asia, and sublineage 2 is seen mainly in isolates recovered in North America. From the timeline, an origin in Asia is possible. However, the absence of isolates from Europe, South America and Africa likely reflects the paucity of WGS data currently available from these continents rather than an absence of GC1 in them, and that fewer GC1 isolates have been sequenced relative to GC2 isolates. Efforts to rectify this deficiency will be needed before the trajectory of these two sublineages can be fully resolved.

## Data bibliography

1. Adams, MD, Chan, ER, Molyneaux, N, Bonomo, RA. NCBI GenBank ADHA01 *A. baumannii* AB058, WGS, ADGZ01 *A. baumannii* AB056, WGS, and ADHB01 *A. baumannii* AB059, WGS (2010).
2. Arivett, BA, Fiester, SE, Ream, DC, Actis, LA. NCBI GenBank LREM01 *A. baumannii* AB4991 (2016).
3. Brinkac, LM, Chopra, S, Jones, MB. NCBI GenBank AUWL01 *A. baumannii* 1605 (2013).
4. Cerqueira, G, Feldgarden, M, Courvalin, P, Perichon, B, Grillot-Courvalin, C, Clermont, D, Rocha, E, Yoon, EJ, Nemec, A, Walker, B, Young, SK, Zeng, Q, Gargeya, S, Fitzgerald, M, Haas, B, Abouelleil, A, Alvarado, L, Arachchi, HM, Berlin, AM, Chapman, SB, Dewar, J, Goldberg, J, Griggs, A, Gujja, S, Hansen, M, Howarth, C, Imamovic, A, Larimer, J, McCowan, C, Murphy, C, Neiman, D, Pearson, M, Priest, M, Roberts, A, Saif, S, Shea, T, Sisk, P, Sykes, S, Wortman, J, Nusbaum, C, Birren, B. NCBI Short Read Archive SRR654201 *A. baumannii* ANC 4097 (2013).
5. Earl, A, Limbago, B, Yoo, B, Ma, P, Battacharyya, R, Hung, D, McCowan, C, Young, S, Abouelleil, A, Bochicchio, J, Cao, P, Chapman, S, Cusick, C, Shea, T, Neafsey, D, Nusbaum, C, Birren, B. NCBI GenBank MPBW01 *A. baumannii* AR_0045 (2016).
6. Eijkelkamp, BA, Stroeher, UH, Hassan, KA, Paulsen, IT, Brown, MH. NCBI GenBank JRWO01 *A. baumannii* 6870155, WGS (2014).
7. Hamidian, M., Adams, M., Venepally, P. and Hall, R. NCBI GenBank CP001182 *A. baumannii* AB0057, complete genome (2017).
8. Hamidian, M., Wick, RR., Hawkey, J., Holt, KE. and Hall, RM. NCBI GenBank CP021782 *A. baumannii* A85, complete genome (2017).
9. Harkins, DM., Durkin, AS., Beck, E., Fedorova, NB., Kim, M., Onuska, J., Radune, D., DePew, J., Koroleva, GI., Singh, I., Chahine, MA., Cash, DM., Huang, X. -Z., Nikolich, MP., Nierman, WC. and Fouts, DE. NCBI Short Read Archive SRR387315 *A. baumannii* Canada-BC1, SRR353953 *A. baumannii* Canada-BC5 and SRR387296 *A. baumannii* IS-58 (2012).
10. Harkins, DM., Lesho, E., Waterman, PE., Chan, A. and Fouts, DE. NCBI GenBank JPHV01 *A. baumannii* MRSN 7339 (2014).
11. Holt, KE., Hamidian, M., Kenyon, JJ., Wynn, MT., Hawkey, J., Pickard, D. and Hall, RM. NCBI GenBank CP010781 *A. baumannii* A1, complete genome (2015).
12. Holt, KE., Hamidian, M., Kenyon, JJ., Wynn, MT., Hawkey, J., Pickard, D. and Hall, RM. European Nucleotide Archive Project ERP001080 *A. baumannii* strains 6772166, RBH3, , D81 and D78, Illumina sequence reads and draft assemblies (2015).
13. Kamolvit, W., Sidjabat, HE. and Paterson, DL. NCBI GenBank LAIO01 *A. baumannii* S36 (2015).
14. Sahl, JW., Driebe, EM., Schupp, JM., Engelthaler, DM. and Keim, P. NCBI GenBank ASFH01 *A. baumannii* TG20277, ASFP01 *A. baumannii* TG22190, ASFR01 *A. baumannii* TG22194, ASFS01 *A. baumannii* TG22196, ASFN01 *A. baumannii* TG22148, ASFK01 *A. baumannii* TG22112 and ASFX01 *A. baumannii* TG22214 (2013).
15. Sahl, JW., Gillece, JD., Schupp, JM., Driebe, EM. and Engenthaler, DM. NCBI GenBank AMHW01 *A. baumannii* 908-13, WGS, AMHZ *A. baumannii* 909-02-7, WGS (2012).
16. The Wellcome Trust Sanger Institute. European Nucleotide Archive Short Read Run accession ERR190502 *A. baumannii* NCSR 106 and ERR190503 *A. baumannii* NCSR 132.
17. Yildimir, SY., Thompson, MG., Harkins, DM., Losada, L., Nierman, W., Zurawski, DV. and Kirkup, BC. NCBI GenBank JHUI01 *A. baumannii* AB5075, WGS (2008).
18. Yoon, E.-J., Kim, JO. and Jeong, SH. NCBI GenBank CP020595 *A. baumannii* USA15 (2017).

## Supplementary Data

Supplementary File 1
