# Supplementary material for: Evolution of a clade of Acinetobacter baumannii global clone 1, lineage 1 via acquisition of carbapenem- and aminoglycoside-resistance genes and dispersion of ISAba1
Source: Microb Genom. 2019 Jan 16;5(1):e000242. doi: 10.1099/mgen.0.000242 (PMC6412058; doi:10.1099/mgen.0.000242)
Supplement: Supplementary File 1 [file mgen-5-242-s001.pdf]

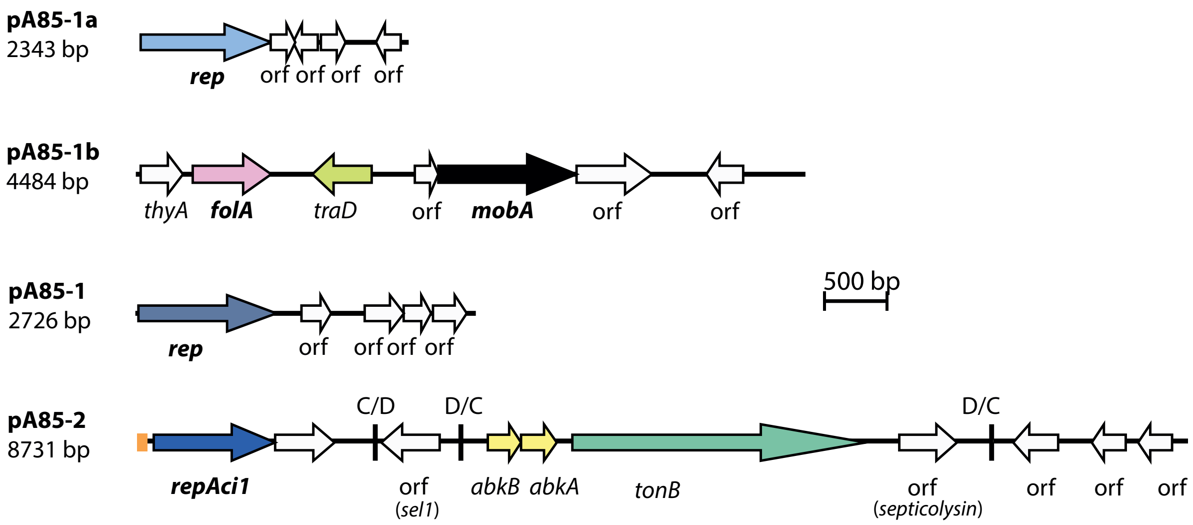

Figure S1. Linearized map of small plasmids found in A85. Thick central lines represent plasmid backbone and horizontal arrows indicate the location, size and orientation of genes/ORFs. Genes encoding replication initiation proteins are shown using different shades of blue. Plasmid maps are drawn to scale using the sequences in GenBank accession numbers CP021783 (pA85-1), CP021784 (pA85-1a), CP021785 (pA85-1b), and CP021786 (pA85-2). The scale bar is also shown.

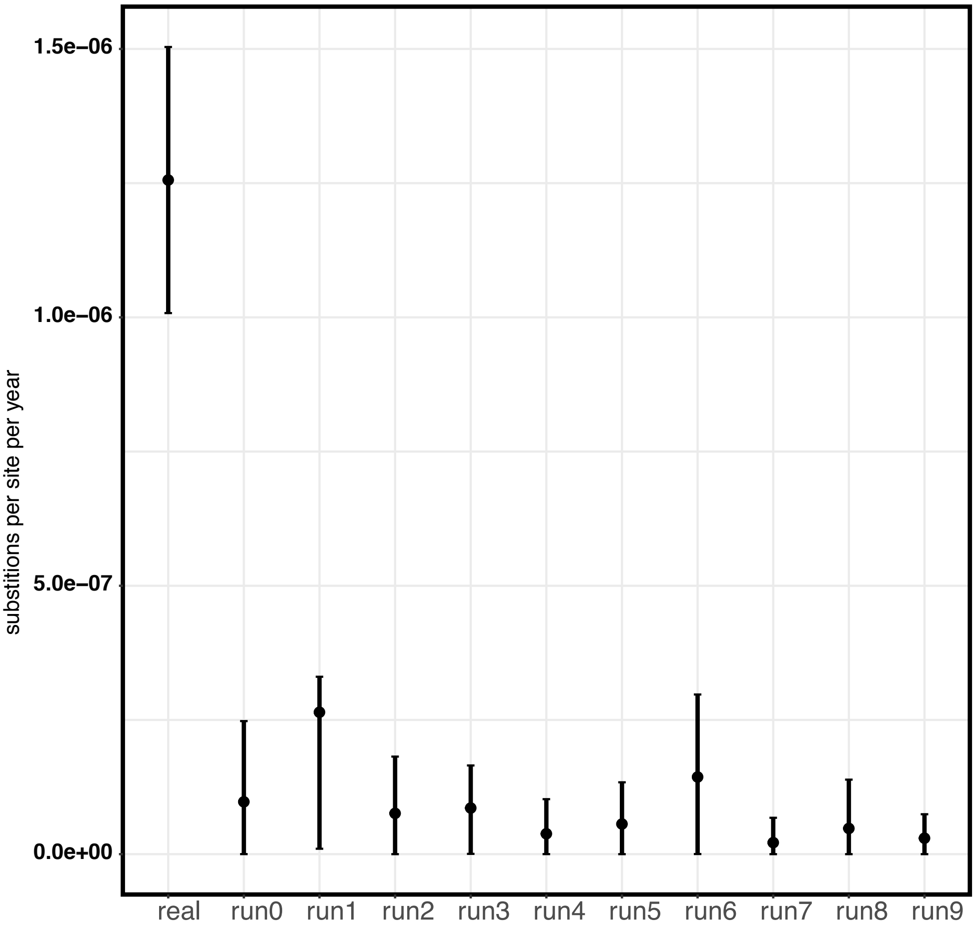

Figure S2. Date randomisation test using BEAST. The vertical bars denote the 95% HPD of substitution rate estimates from the randomisations versus 10 randomisations. The circles denote the mean value. The y-axis is in logarithmic scale.

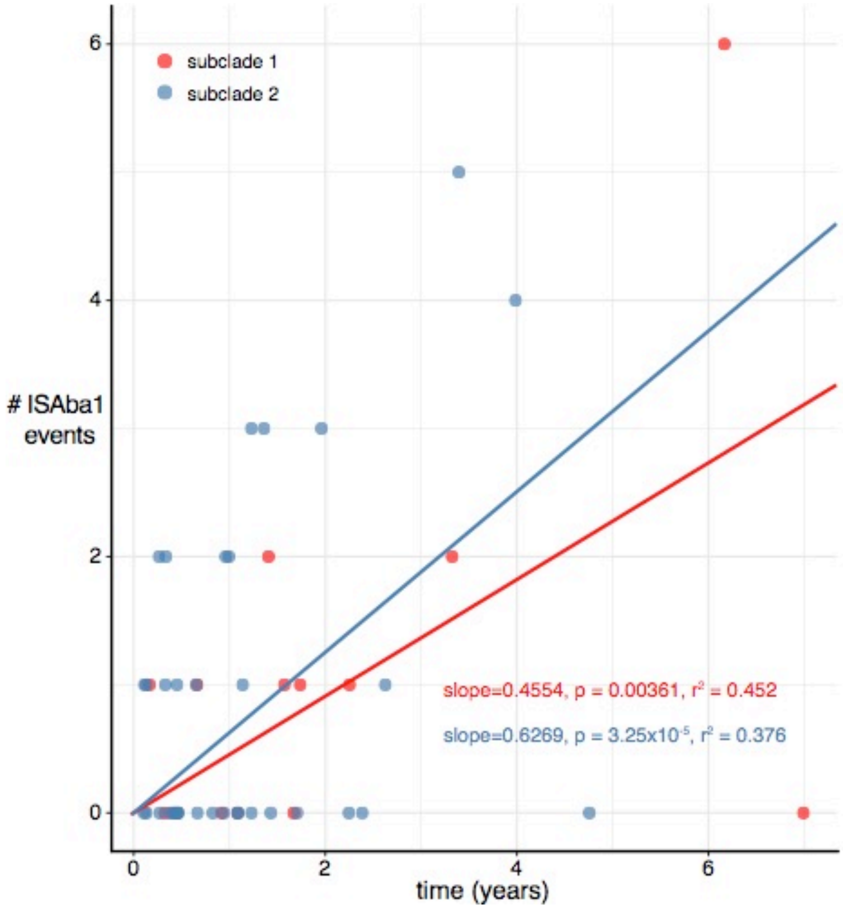

Figure S3. Linear regression of the number of gain events for ISAba1 on each branch of the phylogeny, against the amount of time (in years) each branch represents. Points are coloured by the subclade to which the branch belongs. Regression results are coloured by subclade.

Table S1. Locations of ISAba1 copies [a]

Isolate	Subclade	552960	565014	652225	669156	958076	1112177	1201838	1285633	1443688	1614980	1711640	1737691	1808772	1921072	2331610
A85	1	-	-	-	-	-	+	-	-	-	-	-	-	-	-	-
RBH3	1	-	-	-	-	-	+	-	-	-	-	+	-	-	-	-
6870155	1	-	-	-	-	-	+	-	-	-	-	-	-	-	-	-
6772166	1	-	-	-	-	-	+	-	-	-	-	+	-	-	-	-
S36	1	-	-	-	-	-	+	-	-	-	-	-	-	-	-	-
NCSR_106	1	-	-	+	-	-	+	-	-	-	-	-	-	-	-	-
NCSR_132	1	-	-	+	-	-	+	-	-	-	-	-	-	-	-	-
USA15	1	-	-	+	-	-	+	-	-	-	-	-	-	-	-	-
AB056	2	+	-	-	-	+	-	-	-	-	-	-	-	-	-	-
AB4991	2	+	-	-	-	+	-	-	-	-	-	-	-	-	-	-
MRSN_7339	2	+	-	-	-	+	-	-	-	-	-	-	-	-	-	-
AR_0045	2	+	-	-	-	+	-	-	-	-	-	-	-	-	-	-
AB0057	2	+	-	-	-	+	-	-	-	-	-	-	-	-	-	-
1605	2	+	-	-	-	+	-	+	-	-	+	-	-	-	-	-
TG20277	2	+	-	-	-	+	-	+	-	-	+	-	-	-	-	-
CanadaBC1	2	+	-	-	-	+	-	+	-	-	-	-	+	+	+	-
CanadaBC5	2	+	-	-	-	+	-	+	-	-	-	-	+	+	+	-
AB059	2	+	-	-	-	+	-	-	-	-	-	-	-	-	-	-
ABBL051	2	+	+	-	-	+	-	-	+	-	-	-	-	-	-	-
908_13	2	+	+	-	-	+	-	-	+	+	-	-	-	-	-	-
909_02-7	2	+	+	-	-	+	-	-	+	+	-	-	-	-	-	-
TG22190	2	+	+	-	-	+	-	-	+	+	-	-	-	-	-	-
TG22194	2	+	+	-	+	+	-	-	+	+	-	-	-	-	-	-
TG22196	2	+	+	-	+	+	-	-	+	+	-	-	-	-	-	-
TG22148	2	+	+	-	-	+	-	-	+	+	-	-	-	-	-	+
TG22112	2	+	+	-	-	+	-	-	+	+	-	-	-	-	-	+
TG22214	2	+	+	-	-	+	-	-	+	+	-	-	-	-	-	+

[a] IS at 6670 and 11014 (Tn*6168*) shared by all isolates; numbers represent the 1st base of the 9bp duplication preceeding the IS based on the A85 genome (GenBank no CP021782).

2430451 (Tn2006)	2430451 (Tn2006)	2460567	2739365	2790869	2835637	3864431	Novel IS positions
-	-	-	-	-	-	-	2056113
-	-	+	-	-	-	-	-
-	-	-	-	-	-	-	-
-	-	+	-	-	-	-	-
-	-	-	-	-	-	-	-
-	-	-	-	-	-	-	4033389
-	-	-	-	-	+	-	3365451
-	-	-	-	-	+	-	874526, 6528879, 2546444, 2401479, 1853016 and 139999 (Tn2006)
-	-	-	-	+	-	-	-
-	-	-	-	+	-	-	-
+	+	-	-	+	-	-	-
+	+	-	-	+	-	-	819266
+	+	-	-	+	-	-	-
+	+	-	-	+	-	-	-
+	+	-	-	+	-	-	-
+	+	-	-	+	-	-	-
+	+	-	-	+	-	-	3530524
-	-	-	-	+	-	-	-
-	-	-	-	-	-	+	1349043, 1485056, 3898999, 3125229 and 2616539
-	-	-	-	-	-	+	-
-	-	-	-	-	-	+	-
-	-	-	-	-	-	+	819245, 2849730, 2301158 and 2072909
-	-	-	+	-	-	+	3928959
-	-	-	+	-	-	+	-
-	-	-	-	-	-	+	1310763
-	-	-	-	-	-	+	436681, 257096 and 1435367
-	-	-	-	-	-	+	1847956 and 1538004
